# ACTIGRAPH’S LOW-FREQUENCY EXTENSION FILTER FOR ESTIMATING WRIST-WORN PHYSICAL ACTIVITY IN OLDER ADULTS

**DOI:** 10.1093/geroni/igz038.1918

**Published:** 2019-11-08

**Authors:** Hilary J Hicks, Alex Laffer, Genna Losinski, Amber Watts

**Affiliations:** University of Kansas, Lawrence, Kansas, United States

## Abstract

Advancements in body-worn activity devices make them valuable for objective physical activity measurement. Research-grade monitors utilize software algorithms developed with younger populations using waist-worn devices. ActiGraph offers the low frequency extension (LFE) filter which reduces the movement threshold to capture low acceleration activity that is more common in older adults. It is unclear how this filter changes activity variable calculations in older adults. We investigated the effects of the LFE filter on wrist-worn activity estimates in this population. Participants were 21 older adults who wore the GT9X on their non-dominant wrist for 7 days in a free-living environment. Activity counts were estimated both with and without the LFE filter. Paired samples t-tests revealed that the LFE estimated significantly higher number of counts than non-LFE calculated counts per minute on all three axes (p < .001). Step count estimates were higher with (M = 20,780.09, SD = 5300.85) vs. without (M = 10,896.54, SD = 3489.45) the LFE filter, (t (20) = -22.21, p < .001). These differences have implications for calculations based on axis counts (e.g., Axis-1 calculated steps, intensity level classifications) that rely on waist-worn standards. For example, even without the filter, the GT9X calculated an average of 10,897 steps, which is likely an overestimate in this population. This suggests that axes-based variables should be interpreted with caution when generated with wrist-worn data, and future studies should aim to develop separate wrist and waist-worn standard estimates of these variables in older adult populations.

